# A new strategy for the treatment of heavily pretreated metastatic breast cancer: A case report and review of the literature

**DOI:** 10.1097/MD.0000000000036297

**Published:** 2023-12-01

**Authors:** Ting-Ting Ge, Xiao-Juan Pan, Xi-Meng Zuo, Xiao-Guang Shi, Yu-Kun Wang, Ping Sun, Xiang Gao, Xue Feng, Shuang Gao, Tang-Shun Wang

**Affiliations:** a Department of General Surgery, Dongzhimen Hospital, Beijing University of Chinese Medicine, Beijing, China; b Beijing University of Chinese Medicine, Beijing, China.

**Keywords:** case report, heavily pretreated metastatic breast cancer, utidelone

## Abstract

**Background:**

Breast cancer is one of the most common type of cancers worldwide and remains a critical health issue. Although there are numerous treatment options for advanced metastatic breast cancer, the results are not satisfactory, particularly for triple-negative breast cancer. New treatment modalities need to be explored.

**Case presentation:**

We present the case of a breast cancer patient with multiple metastases who achieved a good response and tolerance to the combination treatment of utidelone plus capecitabine. After being treated with 10 cycles of combined treatment, the patient is now in a good general condition with a progression-free survival time of 10 months.

**Conclusion:**

To our knowledge, this is the first report of utidelone plus capecitabine successfully treating a patient with heavily pretreated metastatic breast cancer. This combined treatment offers a new option for patients with multi-drug resistant breast cancer.

## 1. Introduction

According to the Global Cancer Statistics 2020, female breast cancer is the most prevalent diagnosed tumor in the world.^[1]^ Breast cancer has the highest incidence rate in China, with the number of new cases estimated to be around 410,000 per year, and the number of patients is also increasing yearly. Anthracyclines and taxanes are the first-line drugs for the treatment of breast cancer and are also the drugs of choice for advanced breast cancer. However, due to drug resistance, patients with advanced breast cancer who have received chemotherapy containing paclitaxel or anthracyclines have a certain probability of recurrence of metastasis within 1 year, and those who have failed the second-line treatment after metastasis are considered refractory breast cancer. There is no standard treatment protocol for advanced breast cancer after failing previous multiple lines of therapy. Metastatic breast cancer has a median survival rate of only 15 to 20 months. As a result, the effective treatment of advanced refractory breast cancer has become a challenge and a major issue in the current research field.

Utidelone is an epothilone B derivative that induces apoptosis by promoting microtubule protein polymerization and stabilizing the microtubule structure. In vitro studies have demonstrated that utidelone inhibits the proliferation of breast cancer MCF-7, prostate cancer PC-3, hepatocellular carcinoma HepG2, and multidrug resistant tumor cell lines such as breast cancer NCI/ADR-Res, colon cancer LS1034, and colon cancer HCT15. At the same time, the results of Phase 2 and Phase 3 clinical trials of utidelone indicated its enormous potential in the treatment of heavily pretreated, drug-resistant, and advanced metastatic breast cancer, bringing new hope to patients. We achieved encouraging results in treating patients with advanced breast cancer with utidelone plus capecitabine. The treatment and efficacy of a patient admitted to our hospital with advanced refractory breast cancer are presented below, and the relevant literature is reviewed to further discuss the value of utidelone in patients with advanced metastatic breast cancer. We followed the CARE reporting checklist requirements for case reporting.

## 2. Case presentation

**Figure 1.**
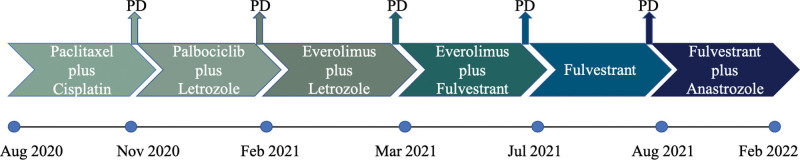
Patient previous treatment timeline and efficacy evaluation.

This study was approved by the Ethics Committee of Dongzhimen Hospital Beijing University of Traditional Chinese Medicine. The patient signed an informed consent. The patient, a 57-year-old female, was admitted to the hospital 21 years after breast-conserving surgery for left breast cancer and 2 years after recurrence. In 2001, the patient found a left breast mass on self-examination and sought treatment at the Cancer Institute and Hospital, Chinese Academy of Medical Sciences. After core needle biopsy, she was diagnosed with breast cancer. On May 8 2001, she underwent breast-conserving surgery for left breast cancer and left axillary lymph node dissection. Pathology examination revealed invasive ductal carcinoma of the breast with a mass size of about 1*1*1 cm and metastatic carcinoma of the lymph nodes (5/18), immunohistochemistry results: ER negative, PR++ positive, CerbB2 negative, P53 negative. Then, she received 6 courses of chemotherapy (Epirubicin and Navelbine) and radiotherapy (50 Gy/25 fractions). After the surgery, she continued taking oral tamoxifen for 5 years. In March 2020, she found a lump, with occasional pain and bleeding on her left breast, but ignored it. As the mass continued to grow, she was admitted at the Sanhuan Cancer Hospital in Chaoyang District, Beijing in August 2020. She then underwent a biopsy of the left breast mass. Unfortunately, the patient was diagnosed with relapsed breast tumor (ER 50% positive, PR negative, Her-2 negative, Ki67 30% positive). The multi-line therapy started from August 2020 (Fig. 1).

**Figure 2.**
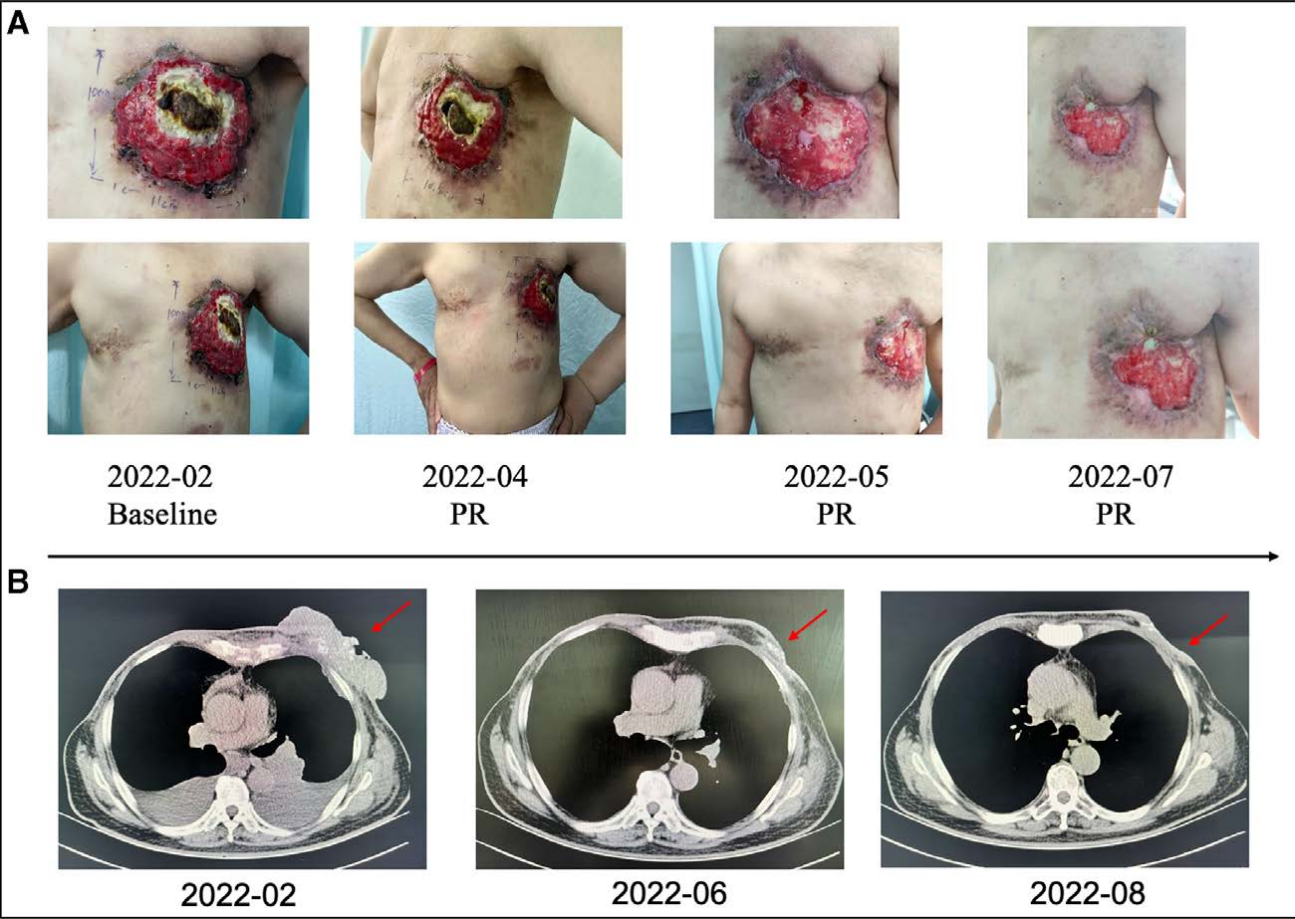
(A) Timeline of left breast tumor changes. Since February 2022, the patient has been receiving a combination treatment of utidelone with capecitabine. There has been a significant reduction in the mass with the progressing treatment cycles. (B) Image changes during combination treatment. The red arrows point to the part of the tumor.

The patient was treated with multiple cycles of chemotherapy and various endocrine drugs but saw poor results, and the breast mass gradually increased in size. In February 2022, the patient was admitted to the Dongzhimen Hospital Beijing University of Traditional Chinese Medicine. The patient was in poor condition and the size of the left breast swelling was about 15*10 cm with multiple metastases. Considering the current tumor load of the patient, she was advised to continue chemotherapy. The patient was observed after admission: a cauliflower-like mass was found in the left breast, with a size of about 15*10 cm, hard texture, with poor mobility, and intermittent bleeding. The patient was diagnosed as stage rT4N3M1 after had a few examinations such as head CT, chest CT, abdominal CT, PET-CT, bone scan. These tests showed she had bone metastasis, no organ metastasis. The first cycle of chemotherapy was performed on February 15, 2022, and the chemotherapy regimen was: utidelone 50 mg D1-D5, capecitabine 2000 mg D1-D14 b.i.d. After 10 cycles of chemotherapy, the tumor markers decreased significantly (Table 1), and remarkable tumor shrinkage was seen (Fig. 2). The patient completed chemotherapy and is now in good general condition with a progression-free survival time of 10 months. Continuous monitoring of routine blood, liver, and kidney function, and electrocardiogram during the treatment period did not reveal any serious adverse events.

**Table 1:** Changes in tumor biomarkers during the patient treatment.

Tumor biomarkers	Time			
	2022-02-07	2022-04-20	2022-07-18	2022-09-16
CEA (ng/ml)	7.97	7.68	6.79	6.06
CA125(U/ml)	576.69	194.31	71.43	48.11
CA153(U/ml)	14.15	9.55	11.41	18.77
CA724(U/ml)	22.41	4.55	2.88	5.44

2022-02-07 test results before starting chemotherapy with utidelone in combination with capecitabine; 2022-04-20 after 3 cycles of chemotherapy; 2022-07-18 after 8 cycles of chemotherapy; 2022-09-16 after 10 cycles of chemotherapy.

## 3. Discussion

In 2001, the patient underwent breast-conserving surgery and axillary lymph node dissection for left-sided breast cancer at the Cancer Hospital of the Chinese Academy of Medical Sciences and received standardized treatment and regular follow-up after surgery. A mass in the left breast was found in March 2020. A fine needle aspiration biopsy was conducted, and it was determined that there was a recurrence of the breast cancer after a 19-year remission period, based on pathological findings. This has been reported in previous literature, and a search reveals relevant reports (Table 2).^[2–5]^

**Table 2:** Case reports of distant recurrent breast cancer metastases.

Case	Clinical presentation	Interval
Xiao L. et al^[2]^	The patient had modified radical surgery mastectomy for right-sided breast cancer in 1991 and received 4 cycles of chemotherapy and tamoxifen for 5 yr after surgery. 2015, she presented with “facial edema and chest distress” and received 18F-FDG-PET/CT, which suggested increased FDG uptake in the right supraclavicular lymph nodes, mediastinum and sternum, and after biopsy, suggested Immunohistochemistry confirmed strong positive ER and PR and positive HER2, and local recurrence of breast cancer was considered.	24 yr
Tashima Y. et al^[3]^	The patient underwent radical surgery for left-sided breast cancer in 1975. 2014, she was seen for lymphedema of the left upper extremity, CT showed soft tissue growth on the left chest wall, and an invasive breast cancer was suggested by biopsy. immunohistochemistry confirmed strong positive ER and PR and negative HER2. Local recurrence of breast cancer was considered.	39 yr
Kusama M. et al^[4]^	The patient underwent modified radical mastectomy for left-sided breast cancer in 1984 and was seen in 2000 for dyspnea, which, in combination with chest CT, suggested pleural metastases.	16 yr
Igarashi T. et al^[5]^	The patient underwent a modified radical mastectomy for breast cancer in 1970, with a case suggestive of a scirrhous carcinoma, and was seen in 1994 for a chest wall tumor, with a recurrence of breast cancer confirmed after puncture.	24 yr

In 1996, Demicheli proposed a bimodal distribution of recurrent metastases after breast cancer surgery, with a peak recurrence at 18 months and 60 months postoperatively, respectively.^[6]^ At 5 years postoperatively, approximately 75% of patients have recurrence. However, a fraction of patients may develop recurrent metastases up to 15 years after surgery. Upon further investigation by Pedersen concerning the risk of breast cancer recurrence beyond 10 years after surgery, it was found that about more than 50% of women with early-stage breast cancer have a 10-eyar tumor-free survival rate, however, there is tumor recurrence after 10 years or more.^[6]^ ER-positive patients have the highest rate of distant recurrence among them. The risk factors for distant recurrence include large tumors, ER (+), positive lymph nodes, and grade I and II tumors.

The distant recurrence of tumors may be related to the dormancy mechanism of tumors. Tumor dormancy is one of the biological features of malignant tumors, and refers to tumor cells that can survive for a long time without exhibiting malignant proliferative characteristics. The main cause of metastatic recurrence is the reactivation of disseminated tumor cells that are closely related to tumor dormancy. Therefore, it is necessary to raise awareness about the risk factors for distant recurrence, and patients with tumor-free status with high-risk recurrence factors should also be followed up for a long time. The mechanism of inhibiting tumor dormancy awakening should be actively investigated to improve the disease-free survival of patients.

In August 2020, the patient underwent biopsy of the left breast mass; the immunohistochemistry results were ER (50%), PR (−), Her2 (1+), and Ki67 (+30%), which was considered estrogen-dependent breast cancer. The patient was subsequently treated with multiple lines of endocrine therapy, but they all resulted with tumor progression as the patient had no significant benefit from endocrine therapy. The 2010 ASCO/CAP guidelines define ER positivity as a percentage of ER positivity ≥ 1% and the guidelines recommend endocrine therapy. In recent years, researchers have discussed the threshold of ER positivity and conducted related studies. It has been argued that patients with ER-positive proportions ranging between 1% and 9% do not benefit from endocrine therapy for breast cancer. Yi et al found in a retrospective study that ER-positive 1% to 9% patients have different clinical and pathological characteristics from ER-positive ≥ 10% patients, and that ER-positive 1% to 9% patients have more similar clinical and pathological characteristics to ER-negative tumors and do not benefit from endocrine therapy.^[7]^ A meta-analysis revealed that patients with ER ratios < 10% did not benefit significantly from adjuvant tamoxifen therapy, but the benefit of adjuvant endocrine therapy increased with increasing ER ratios from a critical value.^[8]^ With continuing research, the 2020 ASCO/CAP guidelines defined ER nuclear staining positive ratios ranging from 1% to 10% as ER weakly positive breast cancer. Fudan University published a propensity-matched study in Cancer in February 2022 comparing survival differences between patients with ER weakly positive early stage breast cancer who did not receive endocrine therapy and patients who received 2 to 3 years or 5 years of endocrine therapy, and ultimately found that there was no significant difference in disease-free survival among patients who received 2 to 3 years and 5 years of endocrine therapy.^[9]^ The majority of current studies focus on the 10% ER threshold. In the current case, the immunohistochemistry of the patient suggested ER-positive breast cancer, however, the patient could not benefit from multiple lines of endocrine therapy, even though the tumor was controlled in later chemotherapy. Thus, whether patients can benefit from endocrine therapy when the ER ratio is <50% is a question that needs further consideration. The patient continued to progress after endocrine therapy for ER-positive breast cancer, and the question of whether chemotherapy should be intervened, as well as the timing of the intervention remains to be considered.

In the current case, we can see that the patient received chemotherapy as well as multiple lines of endocrine therapy, all of which resulted in tumor progression, leading to a treatment dilemma. After trying the combination of utidelone and capecitabine, the patient left breast tumor lesion shrank significantly, and the patient tolerated the treatment well, providing a new option for chemotherapy in advanced breast cancer patients. Currently, chemotherapy remains the cornerstone of treatment for advanced breast cancer, especially HR-positive HER-2 negative advanced breast cancer with visceral crisis; HER-2 positive advanced breast cancer and triple negative breast cancer. In recent years, new advances in drugs targeting chemotherapy for advanced breast cancer have been made, such as the non-paclitaxel microtubule protein inhibitor eribulin and epothilone. In a multicenter, randomized, open, phase III study of eribulin versus vinorelbine for locally recurrent and advanced breast cancer, the median progression-free survival (PFS) was 2.8 months in both the eribulin and vincristine groups, with the eribulin group having a median overall survival (OS) of 13.4 months and the vinorelbine group having a 12.5-month median OS.^[10]^ When compared to the data published for eribulin, the improvement in median PFS and OS for utidelone was clearly superior (Table 3),^[10–12]^ however, more “head-to-head” comparative clinical studies are needed to confirm this.

**Table 3:** Phase II and Phase III clinical studies of Utidelone for advanced metastatic breast cancer.

Researchers	Study Subjects	Interventions	Findings	Safety
Pin Zhang et al^[10]^	18–70 yr of age with advanced metastatic breast cancer; prior chemotherapy regimen including anthracycline-containing and/or paclitaxel-based regimens	33 patients were included in the combination group with utidelone in combined with capecitabine; 63 patients were included in the monotherapy group (utidelone)	ORR of 42.4% and median PFS of 7.9 mo in the combination treatment group; ORR of 28.57% and median PFS of 5.4 mo in the monotherapy group	Common toxicity is peripheral neuropathy and fatigue, with limited and controllable toxicity
Pin Zhang et al^[11]^ Xu B. et al^[12]^	Patients with metastatic breast cancer resistant to anthracycline and paclitaxel-based chemotherapy regimens	270 subjects were included in the utidelone combined with capecitabine group; 135 were included in the capecitabine monotherapy group	The median PFS was 8.44 mo (95% CI 7.95–9.92) in the combination therapy group and 4.55 mo (95% CI 2.55–9.39) in the monotherapy group. The median OS was 19.8 mo in the combination therapy group compared with 16.0 mo in the monotherapy group [(HR) = 0.75, 95% CI 0.59–0.94, *P* = .0142	Common toxicity is peripheral neuropathy

According to the phase II and phase III clinical trial reports on utidelone, patients with metastatic breast cancer benefited significantly from utidelone treatment, with PFS and OS being significantly longer in the combination group of utidelone and capecitabine than in the capecitabine monotherapy group. In addition, we discovered a high safety profile for utidelone in the above study, with the most common adverse effect being peripheral neuropathy, which can be improved by lowering the dose of the drug, or delaying chemotherapy or symptomatic treatment, with a median remission time of about 4 weeks reported for peripheral neuropathy. The efficacy of euthyroid in metastatic breast cancer has been confirmed, and it can significantly improve median PFS and OS, providing a new option for patients with multi-drug resistant breast cancer.

## 4. Conclusion

Utidelone is the first epothilone derivative engineered using a biosynthetic approach that has completed a phase 3 clinical trial. In conclusion, utidelone is a new generation of potential antineoplastic drug that can achieve significant efficacy after resistance to anthracycline and paclitaxel multi-line therapy, and has good safety and controllable adverse effects, and thus has good application value and prospects.

## Acknowledgments

We are particularly grateful to all the people who have given us help on our article.

## Author contributions

**Conceptualization:** Xiao-Juan Pan, Xi-Meng Zuo, Xiao-Guang Shi, Yu-Kun Wang, Xiang Gao.

**Data curation:** Ting-Ting Ge, Xiao-Juan Pan, Xi-Meng Zuo, Ping Sun, Xue Feng, Shuang Gao, Tang-Shun Wang.

**Writing – original draft:** Ting-Ting Ge.

**Writing – review & editing:** Xiao-Guang Shi, Yu-Kun Wang, Ping Sun, Xiang Gao, Xue Feng, Shuang Gao, Tang-Shun Wang.
